# Three-dimensional aneurysm wall enhancement in fusiform intracranial aneurysms is associated with aneurysmal symptoms

**DOI:** 10.3389/fnins.2023.1171946

**Published:** 2023-05-05

**Authors:** Xuge Chen, Fei Peng, Xinmin Liu, Jiaxiang Xia, Hao Niu, Xiaoxin He, Boya Xu, Xiaoyan Bai, Zhiye Li, Peng Xu, Yonghong Duan, Binbin Sui, Xingquan Zhao, Aihua Liu

**Affiliations:** ^1^Beijing Neurosurgical Institute and Beijing Tiantan Hospital, Capital University, Beijing, China; ^2^Department of Neurology, Beijing Tiantan Hospital, Capital Medical University, Beijing, China; ^3^Tiantan Neuroimaging Center of Excellence, China National Clinical Research Center for Neurological Diseases, Beijing, China; ^4^Department of Neurosurgery, The Second Affiliated Hospital, Hengyang Medical School, University of South China, Hengyang, Hunan, China

**Keywords:** intracranial aneurysm, aneurysm wall enhancement, MRI, inflammation, three-dimensional

## Abstract

**Background and purpose:**

Aneurysm wall enhancement (AWE) in high-resolution magnetic resonance imaging (HR-MRI) is a potential biomarker for evaluating unstable aneurysms. Fusiform intracranial aneurysms (FIAs) frequently have a complex and curved structure. We aimed to develop a new three-dimensional (3D) aneurysmal wall enhancement (AWE) characterization method to enable comprehensive FIA evaluation and to investigate the ability of 3D-AWE to predict symptomatic FIA.

**Methods:**

We prospectively recruited patients with unruptured FIAs and received 3 T HR-MRI imaging from September 2017 to January 2019. 3D models of aneurysms and parent arteries were generated. Boundaries of the FIA were determined using 3D vessel diameter measurements. D_max_ was the greatest diameter in the cross-section, while L_max_ was the length of the centerline of the aneurysm. Signal intensity of the FIA was normalized to the pituitary stalk and then mapped onto the 3D model, then the average enhancement (3D-AWE_avg_), maximum enhancement (3D-AWE_max_), enhancement area (AWE_area_), and enhancement ratio (AWE_ratio_) were calculated as AWE indicators, and the surface area of the entire aneurysm (A_area_) was also calculated. Areas with high AWE were defined as those with a value >0.9 times the signal intensity of the pituitary stalk. Multivariable logistic regression analyses were performed to determine independent predictors of aneurysm-related symptoms. FIA subtypes were defined as fusiform, dolichoectasia, and transitional. Differences between the three FIA subtypes were also examined.

**Results:**

Forty-seven patients with 47 FIAs were included. Mean patient age was 55 ± 12.62 years and 74.5% were male. Twenty-nine patients (38.3%) were symptomatic. After adjusting for baseline differences in age, hypertension, L_max_, and FIA subtype, the multivariate logistics regression models showed that 3D-AWE_avg_ (odds ratio [OR], 4.029; *p* = 0.019), 3D-AWE_max_ (OR, 3.437; *p* = 0.022), AWE_area_ (OR, 1.019; *p* = 0.008), and AWE_ratio_ (OR, 2.490; *p* = 0.045) were independent predictors of aneurysm-related symptoms. D_max_ and A_area_ were larger and 3D-AWE_avg_, 3D-AWE_max_, AWE_area_, and AWE_ratio_ were higher with the transitional subtype than the other two subtypes.

**Conclusion:**

The new 3D AWE method, which enables the use of numerous new metrics, can predict symptomatic FIAs. Different 3D-AWE between the three FIA subtypes may be helpful in understanding the pathophysiology of FIAs.

## Introduction

Fusiform intracranial aneurysms (FIAs) account for 3 to 13% of intracranial aneurysms (IAs) (Park et al., 2008). Compared with saccular aneurysms, which account for the majority of IAs, FIAs have no definable neck, and are approximately 1.5 times the diameter of a normal vessel (Flemming et al., 2005). The pathophysiological processes underlying FIAs are complex. FIAs can be classified into fusiform, dolichoectatic, and transitional subtypes, each having a different natural history (Flemming et al., 2005). Inflammation plays a key role in the growth and rupture of IAs (Chalouhi et al., 2012, 2013). Aneurysmal wall enhancement (AWE) on high-resolution magnetic resonance imaging (HR-MRI) is a new imaging biomarker of aneurysmal wall inflammation (Quan et al., 2019) which has been associated with aneurysm-related symptoms and IA instability (Larsen et al., 2018; Samaniego et al., 2019).

In previous histopathological IA studies, AWE correlated with atherosclerosis, neovascularization, and macrophage infiltration (Shimonaga et al., 2018; Hudson et al., 2019). Atherosclerosis may be involved in FIA pathogenesis but the underlying mechanism remains unclear (Park et al., 2008). FIAs may represent a broad regional pathological process because they exhibit greater AWE in a larger vascular area than saccular aneurysms (Nakatomi et al., 2000; Liu et al., 2020; Sabotin et al., 2021). Therefore, a standardized method for quantifying AWE may improve objectivity and reliability for assessing FIA pathophysiology.

Recently, aneurysm-to-pituitary stalk contrast ratio (CR_stalk_) was reported as the most reliable quantitative parameter of AWE (Roa et al., 2020). Growing aneurysms have a significantly higher CR_stalk_ than stable ones (Omodaka et al., 2018). In our previous study, using a CR_stalk_ with the maximum signal intensity (CR_stalk-max_) cut-off value of 0.90 had the highest sensitivity for identifying symptomatic FIAs (Peng et al., 2022). Most previous studies have been based on two-dimensional (2D) manual multiplanar reconstruction selection (Samaniego et al., 2019; Wang et al., 2019), however, the structure of most FIAs is complex with curved surfaces, so the aneurysm and its boundaries are difficult to characterize in just one plane. Therefore, three-dimensional (3D) AWE characterization of FIAs may be more comprehensive and accurate.

We recently developed a 3D AWE characterization method for saccular IAs (Fu et al., 2022). In the current study, we have modified the method for use with FIAs to enable quantitative analysis of AWE with determined boundaries in 3D space. We hypothesized that 3D AWE in FIAs predicts aneurysm-related symptoms and that 3D AWE characteristics differ between the three FIA subtypes.

## Methods

### Study population and data collection

Patients with unruptured FIAs detected by digital subtraction angiography (DSA), computed tomography angiography (CTA), or magnetic resonance angiography (MRA) were prospectively recruited at Beijing Tiantan Hospital from September 2017 to January 2019. Institutional ethics committee approval was obtained. All participants provided written informed consent. Patients with an MRI contraindication, MRI of poor quality, incomplete MRI, history of surgical or endovascular IA treatment, or coexisting saccular IA, vascular dissection, or other intracranial cerebrovascular disease were excluded. Aneurysm-related symptoms were defined as sentinel headache or oculomotor nerve palsy (Fu et al., 2021). Sentinel headache was defined as a sudden severe headache on the same side of the aneurysm during the recent 2 weeks without prior history of headache during the last 5 years, while oculomotor nerve palsy was defined as a sudden headache on the same side of the aneurysm accompanied by one or more symptoms such as disappearance of pupil light reflex, ptosis, or paralysis of the extraocular muscles during the recent 1 month (Fu et al., 2021). Two experienced neuroradiologists (20 and 8 years of experience in neuroradiology, respectively) determined whether the symptoms were related to the fusiform intracranial aneurysm (e.g., symptoms were consistent with the location and size of the FIA) by consensus. As in our previous studies, D_max_ was defined as the greatest diameter in the cross-section (Peng et al., 2022). Specifically, our in-house tool automatically extracts the vessel centerline from the parent artery and aneurysm model based on the feature tree growth algorithm. The fusiform aneurysm was intercepted perpendicular to the centerline to obtain a cross-section, and the maximum diameter in all of the cross-sections was defined as D_max_. L_max_ was defined as the length of the centerline of the fusiform aneurysm (Peng et al., 2022). Thrombus is defined as the intraluminal thrombus of the aneurysm (Cao et al., 2020). Atherosclerosis is defined as eccentric, calcified plaques located in the aneurysm (Sacho et al., 2014). Current smoker is defined as patients who smoked ≥100 cigarettes during the past year (Feng et al., 2018).

### MRI protocol

MRI was performed using a 3.0 T Trio-Tim (Siemens Healthcare, Erlangen, Germany), Ingenia CX (Philips Healthcare, Best, Netherlands), or Discovery 750 (GE Healthcare, Milwaukee, WI, United States) system with a 32-channel head coil. FIAs were localized using 3D time-of-flight MRA. The HR-MRI protocol included 3D T1-weighted imaging (SPACE/VISTA/CUBE), 3D T2/proton density imaging (SPACE/VISTA/CUBE), and contrast-enhanced 3D T1-weighted imaging (SPACE/VISTA/CUBE). Images were acquired in the oblique coronal plane to cover the entire aneurysm. Voxel size was 0.7 × 0.7 × 0.7 mm^3^. Post-contrast T1-weighted images were obtained 6 min after injection of contrast (0.1 mmol/kg gadopentetate dimeglumine [Gd-DTPA]) using parameters identical to those of the pre-contrast T1-weighted images. Other sequence parameters are provided in Supplementary Table 1.

### AWE analysis

**Figure 1:**
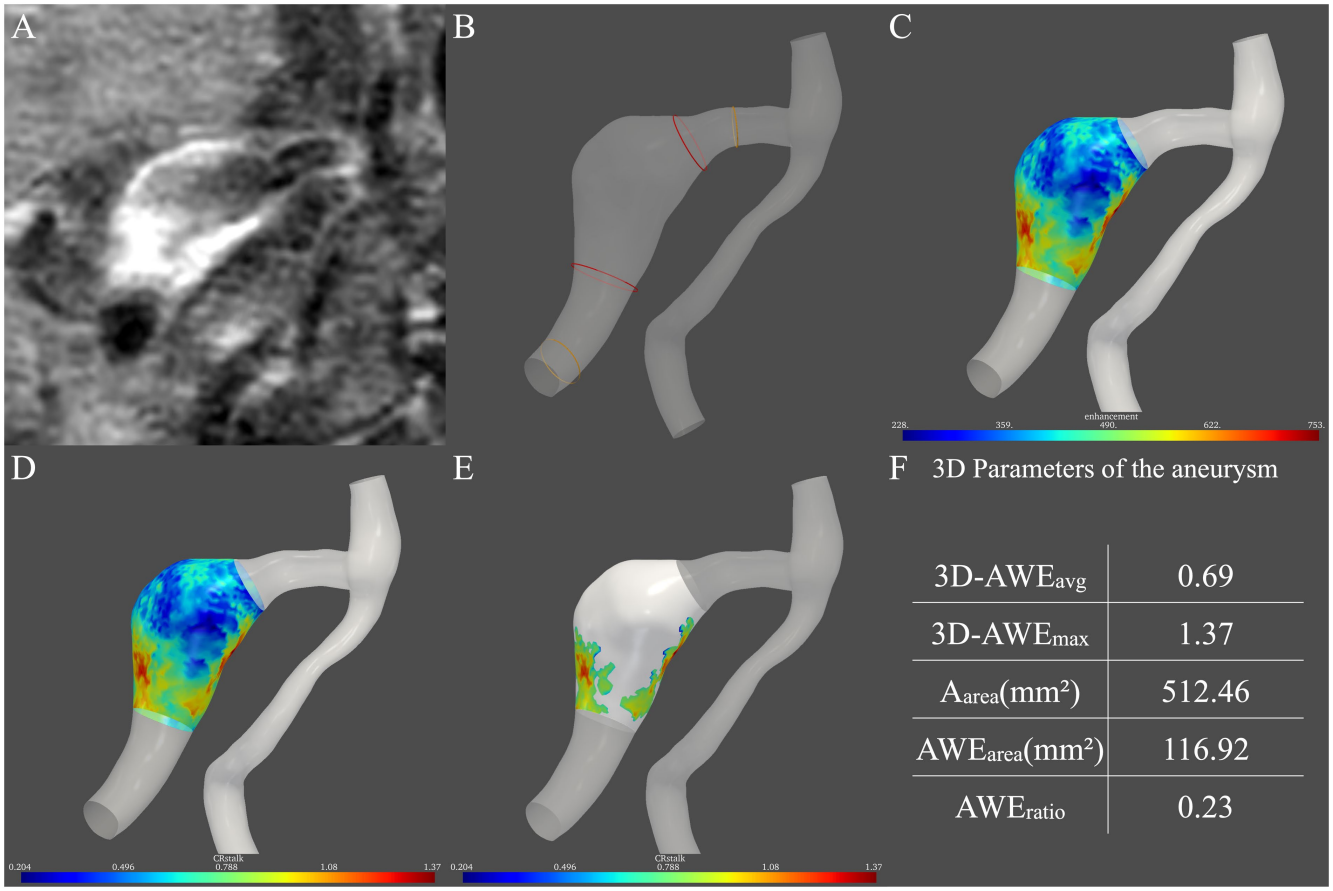
Procedure for measurement of three-dimensional aneurysmal wall enhancement (AWE) on high-resolution magnetic resonance imaging. **(A)** AWE in the two-dimensional plane was first observed. **(B)** Then, the two boundaries of the aneurysm (red ring) were defined as 1.5 times the normal vessel (yellow ring) diameter. **(C)** After the signal intensity (SI) of the post-contrast images was projected onto the model, the SI of the entire aneurysm was obtained. **(D)** Then, CR_stalk_ was calculated using the SI of the entire aneurysm. **(E)** Areas with high AWE (>0.9 × SI_stalk_) were then observed. **(F)** Finally, five indicators in three dimensions were obtained: 3D-AWE_avg_, 3D-AWE_max_, A_area_, AWE_area_, and AWE_ratio_.

We previously described a method for semi-automatic analysis of 3D AWE in FIAs (Fu et al., 2022). In the current study, we aimed to establish an aneurysm model which can obtain both the aneurysm morphology and aneurysm wall enhancement. Based on post-contrast 3D T1-weighted images (Figure 1A), each aneurysm model and the parent artery were generated manually using 3D Slicer (http://www.slicer.org). The boundary of an FIA was defined as 1.5 times the diameter of the normal vessel (Figure 1B), which was measured based on our in-house software. Vessel wall segmentation and identification of aneurysm boundaries were performed by two neuroradiologists with 20 and 15 years of neurovascular imaging experience, respectively. Any disagreements were resolved by consensus.

To quantify the spatial distribution of the signal intensity of the entire aneurysm, the SI of HR-MRI images after contrast administration was projected onto the aneurysm model (Raghuram et al., 2021). An in-house tool programmed by Python mapped the signal intensity on the post-contrast T1 image to the aneurysm model. Specifically, for each vertex on the aneurysm model, probes were created perpendicular to the vertice. The signal intensity on the post-contrast T1 image is sampled on the probes, and the highest signal intensity on each probe is used to determine the signal intensity on the wall. Then, the average signal intensity was taken. Therefore, the distribution of aneurysmal wall enhancement in three dimensions was observed (Figure 1C).

To quantify FIA AWE, the previous 2D-CR_stalk_ was calculated as the contrast ratio of the averaged SI of the aneurysm plane at the level of the maximal aneurysm size to the pituitary stalk (Roa et al., 2020). To compare with 2D-CR_stalk_, the aneurysm plane was replaced by the entire aneurysm. The SI of four points was randomly selected on the pituitary stalk and the mean value of these four points was defined as SI_stalk_, which followed the methods of our previous study (Peng et al., 2022). Specifically, SI_stalk_ was determined by a blinded neuroradiologist with 20 years of neurovascular imaging experience using Horos (https://horosproject.org/). The SI of the aneurysm wall was then normalized to SI_stalk_ to demonstrate 3D-CR_stalk_ (Figure 1D). To identify areas with high AWE, based on our previous FIA study (Peng et al., 2022), a cut-off value of 0.90 was used in 3D-CR_stalk_ to dichotomize high AWE and low AWE, as with 2D-CR_stalk_. Areas with high AWE (> 0.9 × SI_stalk_) were then observed (Figure 1E). Next, we defined and calculated the 3D-AWE parameters of the FIA: 3D-AWE_avg_ was defined as the 3D-CR_stalk_ using the average SI, while 3D-AWE_max_ was defined as the 3D-CR_stalk_ using the maximum SI, and A_area_ was defined as the sum of aneurysm wall areas. To quantify AWE area, AWE_area_ was defined as the sum of aneurysm wall areas with high AWE (>0.9 × SI_stalk_), and AWE_ratio_ was defined as the percentage of AWE area on the surface area of the entire aneurysm (A_area_). Then, using our in-house software, five FIA indicators in three dimensions were calculated: 3D-AWE_avg_, 3D-AWE_max_, A_area_, AWE_area_, and AWE_ratio_ (Figure 1F).

### Statistical analysis

Statistical analyses were conducted using SPSS software version 26 (IBM Corp., Armonk, NY, United States). Variables are expressed as numbers with percentage or medians with interquartile range. Categorical data were compared using Pearson’s chi-square test. Continuous data were compared using the Mann–Whitney test or the Kruskal–Wallis H test. Variables with *p* < 0.10 in univariate analysis were entered into multivariate logistic regression model. To determine 3D-AWE predictors for symptomatic fusiform aneurysms, each 3D-AWE predictor was entered into the multivariate logistic regression models, respectively. In addition, to further identify the best predictor, all of the 3D-AWE_avg_, 3D-AWE_max_, AWE_area_, and AWE_ratio_ were entered into the multivariate logistic regression model. Differences in 3D-AWE between the three FIA subtypes were also investigated. Measurement of AWE was performed by two neuroradiologists with 5 and 10 years of neurovascular imaging experience, respectively. Both were blinded to patient data. Interobserver agreement was evaluated using the intraclass correlation coefficient (ICC) and classified as good (ICC 0.60–0.80) or excellent (ICC >0.80). Interobserver reliability for the CR_stalk_ measurements was assessed using the ICC (two-way random effects, absolute agreement, single rater/measurement form; Koo and Li, 2016). *p* < 0.05 was considered significant.

## Results

**Figure 2:**
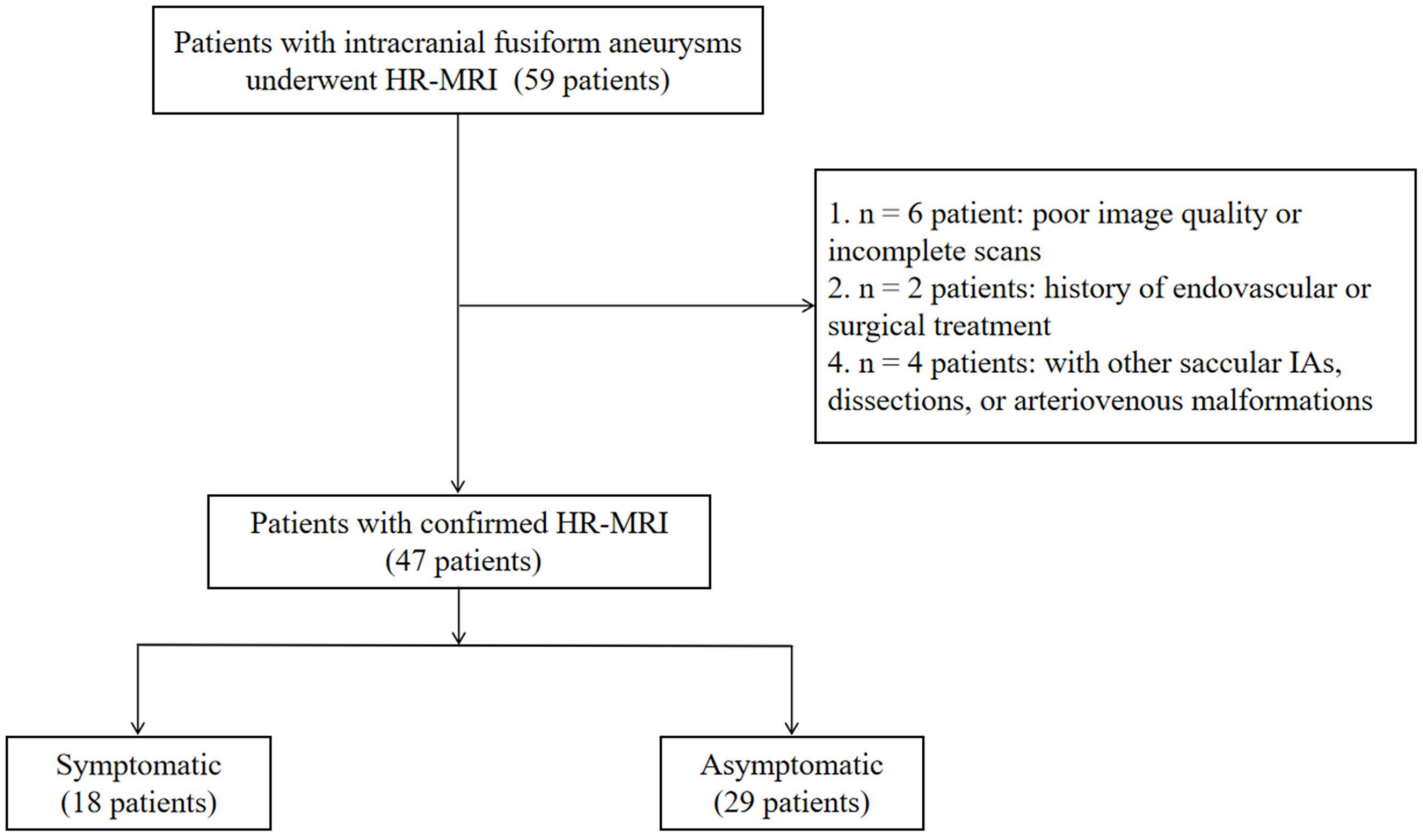
Study flow chart.

Fifty-nine patients with an FIA were recruited. We excluded six with poor quality or incomplete MRI, two with a history of endovascular or surgical aneurysm treatment, and four with other saccular IAs, dissections, or arteriovenous malformations. Finally, 47 patients with 47 FIAs were included for analysis (Figure 2). Mean patient age was 55 ± 12.62 years and 25.5% were female. Twenty-nine patients (61.7%) were asymptomatic. Among the 18 patients with symptoms (38.3%), 15 reported sentinel headache and three had an oculomotor nerve palsy. Among the three FIAs with oculomotor nerve palsy, two locate at the anterior circulation (both are internal carotid fusiform subtype FIAs with D_max_ of 8 mm and 10 mm, respectively) and one locates at the posterior circulation (basilar transitional subtype FIA with D_max_ of 28 mm). FIA subtype was fusiform in 35 patients (74.5%), dolichoectatic in seven (14.9%), and transitional in five (10.6%). Patient characteristics are shown in Table 1.

**Table 1:** Characteristics of patients grouped according to presence of symptoms.

Variable	All, *N* = 47	Symptomatic, *N* = 29	Asymptomatic, *N* = 18	*p*-value
Demographics				
Age	55 (46–64)	51 (37–57)	59 (51–65)	**0.012** ^a^
Female Sex	12 (25.5)	7 (38.9)	5 (17.2)	0.098^b^
Aneurysm location				0.631
Anterior circulation	4 (8.5)	2 (11.1)	2 (6.9)	
Posterior circulation	43 (91.5)	16 (88.9)	27 (93.1)	
Past medical history				
Hypertension	26 (55.3)	6 (33.3)	20 (69.0)	**0.017** ^b^
Diabetes	6 (12.8)	2 (11.1)	4 (13.8)	0.789^b^
Hyperlipemia	21 (44.7)	6 (33.3)	15 (51.7)	0.218^b^
Coronary heart disease	2 (4.3)	0 (0.0)	2 (6.9)	0.255^b^
Thrombus	18 (38.3)	6 (33.3)	12 (41.4)	0.581^b^
Atherosclerosis	25 (53.2)	10 (55.6)	15 (51.7)	0.798^b^
Aspirin	9 (19.1)	2 (11.1)	7 (24.1)	0.270^b^
Statin	14 (29.8)	3 (16.7)	11 (37.9)	0.121^b^
Current smoker	26 (55.3)	9 (50.0)	17 (58.6)	0.563^b^
Clinical scores				
Flemming classification				**0.010** ^a^
Fusiform	35 (74.5)	9 (50.0)	26 (89.7)	
Dolichoectasia	7 (14.9)	5 (27.8)	2 (6.9)	
Transitional	5 (10.6)	4 (22.2)	1 (3.4)	
*FIA characteristics*				
L_max_	15.12 (10.02–21.20)	20.58 (12.62–26.44)	13.00 (10.00–19.61)	**0.022** ^a^
D_max_	7.93 (6.32–9.46)	7.27 (6.56–10.45)	8.00 (6.04–9.33)	0.743^a^
3D-AWE_avg_	0.50 (0.45–0.57)	0.55 (0.49–0.65)	0.46 (0.39–0.53)	**0.001** ^a^
3D-AWE_max_	1.07 (0.91–1.26)	1.23 (1.07–1.37)	1.01 (0.80–1.16)	**0.001** ^a^
AWE_area_	21.73 (4.36–84.87)	62.81 (17.59–101.10)	8.57 (2.10–38.85)	**0.002** ^a^
AWE_ratio_	0.05 (0.02–0.13)	0.11 (0.05–0.19)	0.03 (0.01–0.09)	**0.003** ^a^

Values shown are medians with interquartile range or numbers with percentage unless otherwise indicated. Boldface type indicates statistical significance (*p* < 0.05).

a Kruskal–Wallis test.

b Pearson’s chi-square test.

Median age (*p* = 0.012) and prevalence of hypertension (*p* = 0.017) were significantly higher in the symptomatic group. FIA subtype significantly differed between the groups (*p* = 0.010). L_max_ (13.00 vs. 20.58, *p* = 0.022), 3D-AWE_avg_ (0.46 vs. 0.55, *p* = 0.001), 3D-AWE_max_ (1.01 vs. 1.23, *p* = 0.001), AWE_area_ (8.57 vs. 62.81, *p* = 0.002), and AWE_ratio_ (0.03 vs. 0.11, *p* = 0.003) were significantly higher in the symptomatic group. D_max_ did not significantly differ between the groups (*p* = 0.743). Results of univariate logistics regression for the 3D-AWE parameters are shown in Table 2, we found that 3D-AWE_avg_ [(odds ratio) OR = 4.110, *p* = 0.005], 3D-AWE_max_ [OR = 4.706, *p* = 0.004], AWE_area_ [OR = 1.017, *p* = 0.016], and AWE_ratio_ [OR = 2.853, *p* = 0.025] were all associated with symptomatic FIAs.

**Table 2:** Univariate logistic regression analysis of three-dimensional aneurysmal wall enhancement parameters for prediction of aneurysm-related symptoms.

Variable	Beta (SE)	OR (95%CI)	*P*-value
3D-AWE_avg_	12.869 (4.573)	4.110 (1.536–11.001)	**0.005**
3D-AWE_max_	5.506 (1.909)	4.706 (1.642–13.484)	**0.004**
AWE_area_	0.017 (0.007)	1.017 (1.003–1.031)	**0.016**
AWE_ratio_	9.579 (4.280)	2.853 (1.139–7.145)	**0.025**

SE, standard error; OR, odds ratio; CI, confidence interval.

Boldface type indicates *p* < 0.05.

After adjusting for age, hypertension, L_max_, and FIA subtype, the multivariate logistics regression models showed that 3D-AWE_avg_ (OR = 4.029; *p* = 0.019), 3D-AWE_max_ (OR, = 3.437; *p* = 0.022), AWE_area_ (OR = 1.019; *p* = 0.008), and AWE_ratio_ (OR = 2.490; *p* = 0.045) were independent predictors of aneurysm-related symptoms (Table 3). To further identify the best predictor, all of the 3D-AWE_avg_, 3D-AWE_max_, AWE_area_, and AWE_ratio_ were entered into the multivariate logistic regression model, 3D-AWE_avg_ remained to be the independent predictor (OR = 4.292; *p* = 0.018) of aneurysm-related symptoms (Table 3). Hypertension was also an independent predictor.

**Table 3:** Multivariate logistic regression analysis of three-dimensional aneurysmal wall enhancement parameters for prediction of aneurysm-related symptoms.

Models	Variable	Beta (SE)	Adjusted OR (95% CI)	*p*-value
1	Age, year	−0.023 (0.035)	0.978 (0.912–1.048)	0.520
	Hypertension	−2.234 (0.907)	0.107 (0.018–0.634)	0.014
	L_max_	0.011 (0.050)	1.012 (0.917–1.116)	0.819
	Flemming classification	1.450 (0.673)	4.264 (1.141–15.936)	0.031
	**3D-AWE**_avg_	**1.394 (0.593)**	**4.029 (1.260–12.884)**	**0.019**
2	Age, year	−0.036 (0.035)	0.965 (0.900–1.034)	0.314
	Hypertension	−2.073 (0.882)	0.126 (0.022–0.708)	0.019
	L_max_	0.010 (0.048)	1.010 (0.919–1.110)	0.837
	Flemming classification	1.234 (0.690)	3.435 (0.888–13.289)	0.074
	**3D-AWE**_max_	**1.235 (0.541)**	**3.437 (1.192–9.914)**	**0.022**
3	Age, year	−0.034 (0.033)	0.967 (0.906–1.032)	0.312
	Hypertension	−2.038 (0.781)	0.130 (0.028–0.603)	0.009
	L_max_	0.003 (0.049)	1.003 (0.911–1.104)	0.003
	Flemming classification	0.943 (0.697)	2.567 (0.655–10.068)	0.176
	**AWE**_area_	**0.019 (0.007)**	**1.019 (1.005–1.034)**	**0.008**
4	Age, year	−0.032 (0.034)	0.969 (0.906–1.036)	0.353
	Hypertension	−2.212 (0.871)	0.109 (0.020–0.603)	0.011
	L_max_	0.013 (0.050)	1.014 (0.919–1.118)	0.787
	Flemming classification	1.514 (0.669)	4.546 (1.225–16.873)	0.024
	**AWE**_ratio_	**0.912 (0.455)**	**2.490 (1.022–6.071)**	**0.045**
5	Age, year	−0.017 (0.038)	0.983 (0.914–1.058)	0.651
	Hypertension	−2.278 (0.934)	0.102 (0.016–0.639)	0.015
	L_max_	−0.013 (0.074)	0.988 (0.854–1.142)	0.866
	Flemming classification	2.367 (1.361)	10.663 (0.741–153.456)	0.082
	3D-AWE_max_	0.775 (0.768)	2.170 (0.482–9.778)	0.313
	AWE_area_	−0.225 (0.853)	0.798 (0.150–4.248)	0.792
	AWE_ratio_	−1.343 (1.260)	0.261 (0.022–3.087)	0.287
	**3D-AWE**_avg_	**1.457 (0.618)**	**4.292 (1.279–14.405)**	**0.018**

SE, standard error; OR, odds ratio; CI, confidence interval.

Parameters differentiating three different FIAs were also investigated. Aneurysm-related symptoms (*p* = 0.010), L_max_ (*p* < 0.001), D_max_ (p = 0.004), 3D-AWE_avg_ (p = 0.005), A_area_ (p = 0.001), AWE_area_ (p = 0.002), and AWE_ratio_ (*p* = 0.049) significantly differed between the three FIA subtypes (Table 4). Notably, D_max_, and A_area_ were larger and 3D-AWE_avg_, 3D-AWE_max_, AWE_area_, and AWE_ratio_ were higher with the transitional subtype than the other two. Dolichoectasia type FIAs showed higher L_max_ [p = 0.022].

**Table 4:** Patient characteristics and imaging parameters according to aneurysm subtype.

Variable	Fusiform, N = 35	Dolichoectasia, N = 7	Transitional, N = 5	*p*-value
Demographics				
Age	57 (50–65)	49 (41–53)	54 (34–66)	0.147^a^
Female Sex	9 (25.7)	1 (14.3)	2 (40.0)	0.601^b^
Aneurysm location				0.473^b^
Anterior circulation	4 (11.4)	0 (0.0)	0 (0.0)	
Posterior circulation	31 (88.6)	7 (100.0)	5 (100.0)	
Past medical history				
Hypertension	20 (57.1)	3 (42.9)	3 (60.0)	0.767^b^
Diabetes	5 (14.3)	1 (14.3)	0 (0.0)	0.664^b^
Hyperlipemia	17 (48.6)	3 (42.9)	1 (20.0)	0.483^b^
Coronary heart disease	2 (5.7)	0 (0.0)	0 (0.0)	0.699^b^
Thrombus	13 (37.1)	1 (14.3)	4 (80.0)	0.067^b^
Atherosclerosis	16 (45.7)	4 (57.1)	5 (100.0)	0.073^b^
Aspirin	8 (22.9)	1 (14.3)	0 (0.0)	0.449^b^
Statin	12 (34.3)	2 (28.6)	0 (0.0)	0.292^b^
Current smoker	17 (48.6)	6 (85.7B27)	3 (60.0)	0.192^b^
FIA characteristics				
Symptomatic	9 (25.7)	5 (71.4)	4 (80.0)	**0.010** ^b^
L_max_	12.63 (10.00–17.00)	28.14 (26.25–41.54)	20.10 (20.00–28.50)	**<0.001** ^a^
D_max_	7.14 (5.88–9.00)	9.93 (6.58–11.81)	10.82 (9.00–15.00)	**0.004** ^a^
3D-AWE_avg_	0.49 (0.42–0.56)	0.51 (0.48–0.60)	0.54 (0.51–0.66)	0.132^a^
3D-AWE_max_	1.02 (0.84–1.18)	1.12 (0.99–1.26)	1.44 (1.30–1.49)	**0.005** ^a^
A_area_	338.05 (158.68–497.08)	746.11 (686.33–784.07)	1105.64 (569.15–1262.40)	**0.001** ^a^
AWE_area_	11.40 (2.41–31.94)	87.33 (9.11–96.41)	101.03 (69.78–273.84)	**0.002** ^a^
AWE_ratio_	0.04 (0.01–0.10)	0.07 (0.04–0.14)	0.13 (0.10–0.24)	**0.049** ^a^

Values shown are medians with interquartile range or numbers with percentage unless otherwise indicated. Boldface type indicates statistical significance (*p* < 0.05).

a Kruskal–Wallis test.

b Pearson’s chi-square test.

**Figure 3:**
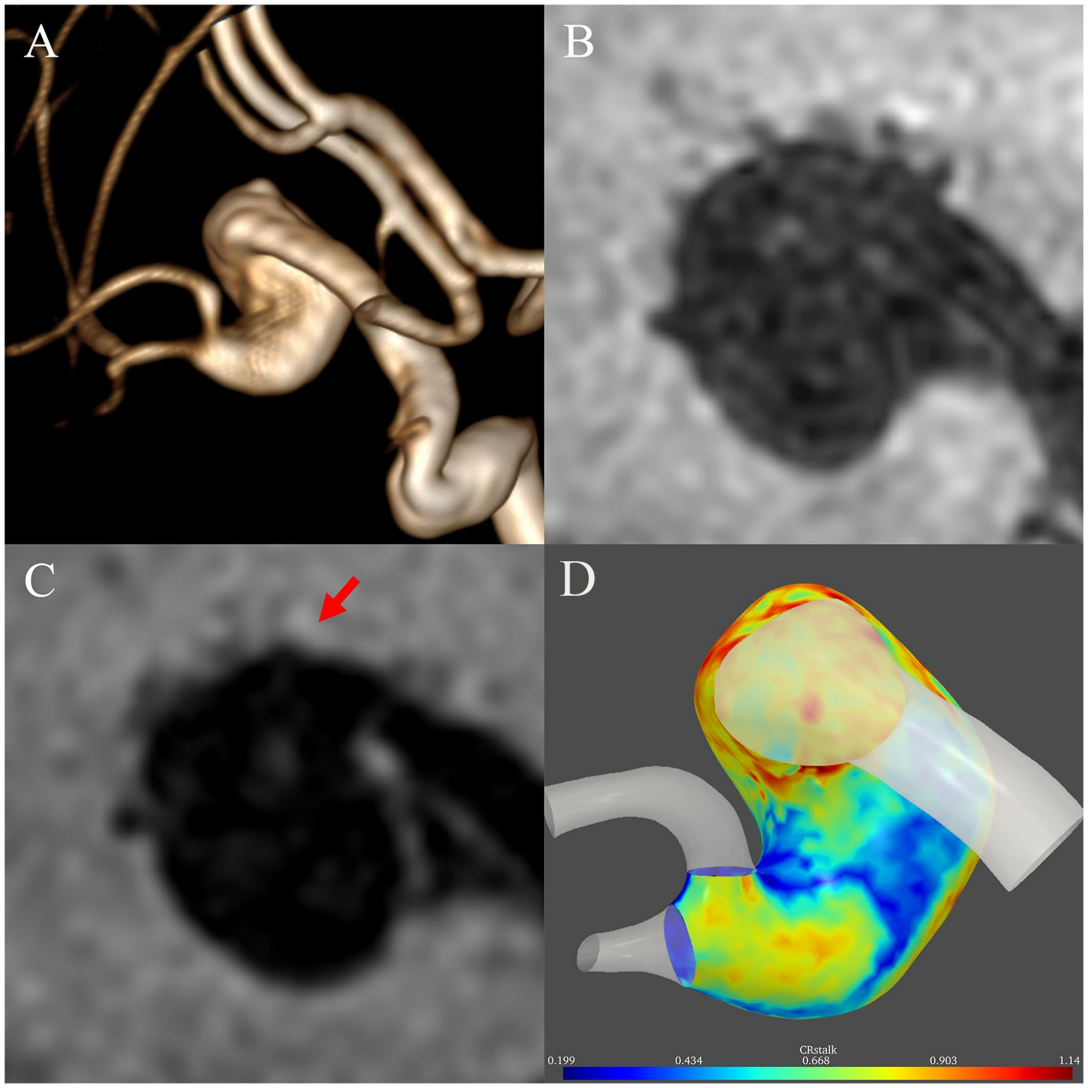
Illustrative case comparing two-dimensional and three-dimensional aneurysmal wall enhancement (AWE). **(A)** A left middle cerebral artery fusiform subtype fusiform intracranial aneurysm with a rotational and distorted structure in three-dimensional space was identified in a 45-year-old man on magnetic resonance angiography. On two-dimensional non-contrast **(B)** and post-contrast **(C)** imaging, AWE can be only seen in one section. In contrast, the three-dimensional imaging **(D)** demonstrated AWE of the entire aneurysm.

Figure 3 presents a 45-year-old man with a left middle cerebral artery fusiform subtype FIA to demonstrate the difference between 2D and 3D AWE. The structure of the aneurysm has rotation and distortion in 3D space. While AWE can be only seen in one section on the 2D view, AWE of the entire aneurysm can be seen on the 3D view.

### Reproducibility of measurements

Interobserver agreement was excellent for the measurement of 3D-AWE_avg_ (ICC, 0.86; 95% confidence interval, 0.78–0.92) and 3D-AWE_max_ (ICC, 0.92; 95% confidence interval, 0.87–0.95).

## Discussion

Because the pathophysiology of FIAs is quite complex and differs from that of saccular IAs, they require a different standardized method for quantifying AWE. In this study, we developed a quantitative assessment protocol for FIAs that can observe AWE distribution in 3D space. We found that FIA 3D AWE is associated with aneurysm-related symptoms and that transitional subtype FIAs tended to be symptomatic, larger, and exhibit greater 3D AWE.

Inflammation of the aneurysm wall is a key factor in the growth and rupture of aneurysms (Meng et al., 2014; Turjman et al., 2014). Furthermore, intracranial AWE on HR-MRI correlates well with aneurysm wall inflammation (Larsen et al., 2018). Therefore, AWE is a potential imaging biomarker for unstable aneurysms. Most studies reporting quantification of AWE have been based on 2D planar imaging. Cao et al. studied the AWE features of vertebrobasilar artery nonsaccular aneurysms and found that enhancement ratio using a cut-off value of 0.8 was an independent predictor of aneurysm-related symptoms (Cao et al., 2020). In our previous study (Peng et al., 2022), CR_stalk-max_ had the highest sensitivity for identification of symptomatic FIAs when a cut-off value of 0.90 was used. However, the pathophysiology of FIAs is complex (Nasr et al., 2018) and predicting their stability may require a comprehensive and objective approach based on 3D space. Current research on 3D-AWE in FIAs is still limited (Raghuram et al., 2022). In this study, we extracted the SI on the T1-weighted sequence using probes perpendicular to the aneurysm wall. To compare with CR_stalk_, the most reliable objective 2D AWE parameter, FIA enhancement was objectively quantified, visualized, and normalized using the pituitary stalk SI. Then, four 3D-AWE parameters were proposed: 3D-AWE_avg_ quantified the overall level of enhancement on the aneurysm wall; 3D-AWE_max_ quantified the highest degree of focal AWE on the aneurysm wall; AWE_area_ was considered a quantification of enhanced aneurysm wall area; and AWE_ratio_ was obtained by normalizing the total area of the aneurysm wall. These attempts may not only be convenient for comparison between different aneurysms but also may be conducive to the classification and quantification of AWE in the entire aneurysm. Raghuram et al. proposed a method to quantify AWE using histograms and 3D heatmaps and reported that FIAs have more focal enhancement areas (Raghuram et al., 2022). In contrast, we defined the area with SI > (SI_stalk_ × 0.9) as the high AWE area for quantifying focal enhancement because (SI_stalk_ × 0.9) is the cutoff value that discriminates aneurysm-related symptoms. We also found that high AWE area independently associated with symptomatic FIAs. Such associations may indicate that more extensive aneurysmal wall inflammation may underly aneurysm-related symptoms, as AWE is considered a biomarker of aneurysm inflammation (Quan et al., 2019). Considering AWE on HR-MRI correlates well with aneurysm wall inflammation (Larsen et al., 2018; Quan et al., 2019), different AWE parameters may demonstrate different degrees or ranges of aneurysm wall inflammation. Therefore, 3D-AWE_max_ may demonstrate the most severe inflammatory area of the aneurysm wall. AWE_area_ may indicate total areas with severe aneurysm wall inflammation. While AWE_ratio_ may reflect the proportion of areas with severe aneurysm wall inflammation, or to say, the burden of aneurysm wall inflammation. Therefore, these parameters may provide a more comprehensive evaluation and prediction of aneurysmal symptoms, aneurysm stability, and risk of rupture than traditional factors, although they need further validation.

Notably, Gd-DTPA enhancement for arterial wall was none specific for inflammation. Fusiform aneurysms often incorporate intraluminal thrombus or atherosclerotic plaque. Meanwhile, FIAs often present complex morphological (thickness) and hemodynamic characteristics, which may affect the enhancement of the aneurysm wall (Sabotin et al., 2021). In this study, 18 (38.3%) FIAs presented with thrombus and 25 (53.2%) FIAs presented with atherosclerosis. The complex morphology of the FIAs may be associated with nonuniform hemodynamics, for example, slow turbulent flow may occur where there are focal widening of the aneurysm, which cause low wall shear stress and promote aneurysm wall inflammation (Cao et al., 2020).

Even using multiplanar reconstruction, AWE analysis in 2D space may not sufficiently reflect 3D structure. First, tracking AWE in one plane may miss focal enhancement, leading to an underestimation of enhancement level. Second, some FIAs are morphologically distorted and their AWE cannot be characterized in a single plane. Third, in 2D planar analysis, regions of interest (ROIs) are typically determined by visual inspection and manual delineation of HR-MRI sequences, which may introduce selection bias and decrease repeatability. Our 3D-AWE protocol enables objective quantitative analysis of the entire aneurysm wall, which may assess FIA more comprehensively and reproducibly and contribute to a better understanding of the pathophysiological processes underlying FIAs. The 3D-AWE protocol in this study has shown its potential for predicting aneurysm-related symptoms. Symptomatic FIAs tended to exhibit a greater level of AWE and larger areas of high AWE, which shows that symptoms may indicate greater aneurysmal wall inflammation. In addition, wall areas that enhance in 3D space may be relatively weak. Therefore, the 3D-AWE protocol described here may help stratify the risk of FIA patients in clinical practice.

Determining FIA boundaries based on 2D imaging is difficult. Flemming et al. defined the FIA boundary as arterial dilation greater than 1.5 times the normal diameter (Flemming et al., 2005). Vessel diameter measurement may have errors when performed in the 2D plane, especially if the measurement plane is not perpendicular to the vessel or the vessel section is not circular. In contrast, vessel centerline and diameter extraction based on the 3D FIA mask can provide diameter measurement of the entire segment containing both the FIA and parent artery, which is helpful to determine FIA boundaries more objectively and accurately. The measurement of vessel centerline length can also more accurately reflect the length of the FIA and generate more reliable 3D morphological parameters in future studies.

Different FIA subtypes have significant differences in the mode of enhancement, which may be due to their different pathological processes (Nasr et al., 2016). In this study, the dolichoectasia subtype had the longest L_max_ and the transitional subtype had the largest D_max_. In addition, it is reported that hemodynamics were also differentiated by different FIA subtypes (Sabotin et al., 2021). As per AWE, AWE_area_ and AWE_ratio_ were all significantly higher in the transitional subtype than the other two subtypes, which indicates the transitional subtype may have more significant aneurysmal wall inflammation. As reported in an imaging follow-up study, the transitional subtype was found to be an independent predictor of instability (Nasr et al., 2016). Therefore, the transitional subtype FIA may grow and rupture more easily (Mangrum et al., 2005). In contrast, the dolichoectasia subtype has a lower risk of rupture than the fusiform subtype or transitional subtype (Mangrum et al., 2005; Passero et al., 2005; Passero and Rossi, 2008), but is significantly longer and may involve more areas of the culprit artery. This may be because its pathological processes markedly differ from those of the fusiform or transitional subtypes (Del Brutto et al., 2021). As we can see, there are significantly different pathological processes among the three FIA subtypes.

### Strengths and limitations

This is the first study to accurately obtain the AWE characteristics of FIAs based on 3D space. In future studies, 3D AWE parameters may provide more information regarding aneurysmal wall inflammation when investigating the associations between AWE and smoking, anti-inflammatory drug use, and other factors. The study has several limitations, however. First, the sample size was small and it was conducted in a single center. With the limited sample size (N = 47), the multivariate logistic model allows for 3 to 4 variables. However, the multivariate logistic model has incorporated 5 to 8 variables, so the statistical model may not allow for so many variables, which is a major statistical concern. Future large-scale multicenter studies are warranted. Second, three different MRI scanners were used, which may have introduced bias and need further validation of the comparability in the future, although the parameters were adjusted consistently. Third, more complex 3D-AWE parameters are needed in the future to explore the pathophysiological mechanisms underlying the different FIA subtypes. Fourth, there may be some limitations about this new 3D-AWE model to evaluate fusiform intracranial aneurysms, such as inadequate spatial resolution, intramural hemorrhage, aneurysm wall thickness, slow-flow, and further pathological validation is required in future studies. Fifth, each aneurysm model was based on post-contrast T1 images, which may introduce some deviations in morphology measurement. Sixth, at bifurcations, normal arteries would be excluded by manual segmentation, which may also cause potential bias. Seventh, because the highest signal intensity on each probe is used to determine the signal intensity on the wall, 3D-AWE in this model may exaggerate the AWE degree and lead to a high false positive rate. Eighth, sentinel headache is more suitable for the warning symptom of saccular aneurysms, and there may be false positives in the diagnosis of symptomatic fusiform aneurysms in this study. Ninth, “enhancement” indicates the signal intensity change after the injection of contrast, while the current study only included post-contrast T1 images. If the aneurysm wall has high signal intensity on the pre-contrast T1 images, the high intensity on the post-contrast images cannot surely indicate “enhancement.” Among all the cases included, there are only 2 aneurysms presented with high signal intensity on pre-contrast images (4.3%, 2 in 47). Although the incidence is low, future studies should incorporate the pre-contrast images. Finally, this study did not exclude the patients withaspirin/stain use, which may suppress the aneurysms wall enhancement (Roa et al., 2020; Kang et al., 2022).

## Conclusion

3D-AWE can predict aneurysm-related symptoms in patients with an FIA. The transitional subtype FIA is associated with a larger cross-sectional size and higher AWE and may grow and rupture more easily than the fusiform and dolichoectasia subtypes. This new AWE analysis method is more accurate and enables use of numerous new metrics which can provide more detailed information for assessing FIA pathophysiology.

## Data availability statement

The raw data supporting the conclusions of this article will be made available by the authors, without undue reservation.

## Ethics statement

The studies involving human participants were reviewed and approved by Beijing Tiantan Hospital Institutional ethics committee. The patients/participants provided their written informed consent to participate in this study. Written informed consent was obtained from the individual(s) for the publication of any potentially identifiable images or data included in this article.

## Author contributions

FP and XC: conception and design. XL, JX, HN, XH, BX, XB, ZL, and PX: acquisition of data. YD and BS: analysis and interpretation of data. XC and FP: drafting the article. XC: technical supports. XZ and AL: study supervision. All of the authors approved the current version to submit.

## Funding

This current study was supported by the National Natural Science Foundation of China (Nos. 82171290 and 81771233), Natural Science Foundation of Beijing Municipality (Nos. 7222050 and L192013), Beijing Municipal Administration of Hospitals’ Ascent Plan (DFL20190501), and Horizontal Project in Beijing Tiantan Hospital (HX-A-027 [2021]), and Research and Promotion Program of Appropriate Techniques for Intervention of Chinese High-risk Stroke People (GN-2020R0007), and BTH Coordinated Development—Beijing Science and Technology Planning Project (Z181100009618035), and Beijing Municipal Administration of Hospitals’ Ascent Plan (DFL20190501), and Beijing Natural Science Foundation (L192013 and 22G10396).

## Conflict of interest

The authors declare that the research was conducted in the absence of any commercial or financial relationships that could be construed as a potential conflict of interest.

## Publisher’s note

All claims expressed in this article are solely those of the authors and do not necessarily represent those of their affiliated organizations, or those of the publisher, the editors and the reviewers. Any product that may be evaluated in this article, or claim that may be made by its manufacturer, is not guaranteed or endorsed by the publisher.

## Supplementary material

The Supplementary material for this article can be found online at: https://www.frontiersin.org/articles/10.3389/fnins.2023.1171946/full#supplementary-material

## References

[ref1] Cao L. Zhu C. Eisenmenger L. du X. Liu J. Yang Q.. (2020). Wall enhancement characteristics of vertebrobasilar nonsaccular aneurysms and their relationship to symptoms. Eur. J. Radiol. 129:109064. doi: 10.1016/j.ejrad.2020.109064, PMID: 32474380

[ref2] Chalouhi N. Ali M. S. Jabbour P. M. Tjoumakaris S. I. Gonzalez L. F. Rosenwasser R. H.. (2012). Biology of intracranial aneurysms: role of inflammation. J. Cereb. Blood Flow Metab. 32, 1659–1676. doi: 10.1038/jcbfm.2012.84, PMID: 22781330 PMC3434628

[ref3] Chalouhi N. Hoh B. L. Hasan D. (2013). Review of cerebral aneurysm formation, growth, and rupture. Stroke 44, 3613–3622. doi: 10.1161/STROKEAHA.113.002390 24130141

[ref4] Del Brutto V. J. Gutierrez J. Goryawala M. Z. Sacco R. L. Rundek T. Romano J. G. (2021). Prevalence and clinical correlates of intracranial Dolichoectasia in individuals with ischemic stroke. Stroke 52, 2311–2318. doi: 10.1161/STROKEAHA.120.032225 33980042 PMC8238812

[ref5] Feng X. Qian Z. Zhang B. Guo E. Wang L. Liu P.. (2018). Number of cigarettes smoked per day, smoking index, and intracranial aneurysm rupture: a case–control study. Front. Neurol. 9:e00380. doi: 10.3389/fneur.2018.00380, PMID: 29904368 PMC5990590

[ref6] Flemming K. D. Wiebers D. O. Brown R. D. Link M. J. Huston J. McClelland R. L.. (2005). The natural history of Radiographically defined Vertebrobasilar Nonsaccular intracranial aneurysms. Cerebrovasc. Dis. 20, 270–279. doi: 10.1159/000087710, PMID: 16123548

[ref7] Fu M. Peng F. Zhang M. Chen S. Niu H. He X.. (2022). Aneurysmal wall enhancement and hemodynamics: pixel-level correlation between spatial distribution. Quant. Imaging Med. Surg. 12, 3692–3693704. doi: 10.21037/qims-21-1203, PMID: 35782262 PMC9246729

[ref8] Fu Q. Wang Y. Zhang Y. Zhang Y. Guo X. Xu H.. (2021). Qualitative and quantitative wall enhancement on magnetic resonance imaging is associated with symptoms of unruptured intracranial aneurysms. Stroke 52, 213–222. doi: 10.1161/STROKEAHA.120.029685, PMID: 33349014 PMC7770055

[ref9] Hudson J. S. Zanaty M. Nakagawa D. Kung D. K. Jabbour P. Samaniego E. A.. (2019). Magnetic resonance vessel wall imaging in human intracranial aneurysms. Stroke 50:e1. doi: 10.1161/STROKEAHA.118.023701 30580739

[ref10] Kang H. Tian D.-C. Yang X. Zhang Y. Li W. Sui B.. (2022). A randomized controlled trial of statins to reduce inflammation in Unruptured cerebral aneurysms. JACC Cardiovasc. Imaging 15, 1668–1670. doi: 10.1016/j.jcmg.2022.04.006, PMID: 36075628

[ref11] Koo T. K. Li M. Y. (2016). A guideline of selecting and reporting Intraclass correlation coefficients for reliability research. J. Chiropr. Med. 15, 155–163. doi: 10.1016/j.jcm.2016.02.012, PMID: 27330520 PMC4913118

[ref12] Larsen N. von der Brelie C. Trick D. Riedel C. H. Lindner T. Madjidyar J.. (2018). Vessel wall enhancement in unruptured intracranial aneurysms: an indicator for higher risk of rupture? High-resolution MR imaging and correlated histologic findings. Am. J. Neuroradiol. 39, 1617–1621. doi: 10.3174/ajnr.A5731, PMID: 30026386 PMC7655285

[ref13] Liu X. Zhang Z. Zhu C. Feng J. Liu P. Kong Q.. (2020). Wall enhancement of intracranial saccular and fusiform aneurysms may differ in intensity and extension: a pilot study using 7-T high-resolution black-blood MRI. Eur. Radiol. 30, 301–307. doi: 10.1007/s00330-019-06275-9, PMID: 31218429

[ref14] Mangrum W. I. Huston J. Link M. J. Wiebers D. O. McClelland R. L. Christianson T. J. H.. (2005). Enlarging vertebrobasilar nonsaccular intracranial aneurysms: frequency, predictors, and clinical outcome of growth. J. Neurosurg. 102, 72–79. doi: 10.3171/jns.2005.102.1.0072, PMID: 15658099

[ref15] Meng H. Tutino V. M. Xiang J. Siddiqui A. (2014). High WSS or low WSS? Complex interactions of hemodynamics with intracranial aneurysm initiation, growth, and rupture: toward a unifying hypothesis. Am. J. Neuroradiol. 35, 1254–1262. doi: 10.3174/ajnr.A3558, PMID: 23598838 PMC7966576

[ref16] Nakatomi H. Segawa H. Kurata A. Shiokawa Y. Nagata K. Kamiyama H.. (2000). Clinicopathological study of intracranial fusiform and Dolichoectatic aneurysms. Stroke 31, 896–900. doi: 10.1161/01.STR.31.4.896, PMID: 10753995

[ref17] Nasr D. M. Brinjikji W. Rouchaud A. Kadirvel R. Flemming K. D. Kallmes D. F. (2016). Imaging characteristics of growing and ruptured Vertebrobasilar non-Saccular and Dolichoectatic aneurysms. Stroke 47, 106–112. doi: 10.1161/STROKEAHA.115.011671, PMID: 26604246

[ref18] Nasr D. M. Flemming K. D. Lanzino G. Cloft H. J. Kallmes D. F. Murad M. H.. (2018). Natural history of Vertebrobasilar Dolichoectatic and fusiform aneurysms: a systematic review and Meta-analysis. Cerebrovasc. Dis. 45, 68–77. doi: 10.1159/000486866, PMID: 29439265

[ref19] Omodaka S. Endo H. Niizuma K. Fujimura M. Inoue T. Endo T.. (2018). Circumferential wall enhancement in evolving intracranial aneurysms on magnetic resonance vessel wall imaging. J. Neurosurg. 131, 1262–1268. doi: 10.3171/2018.5.JNS18322, PMID: 30485237

[ref20] Park S. H. Yim M. B. Lee C. Y. Kim E. Son E. I. (2008). Intracranial fusiform aneurysms: it is pathogenesis, clinical characteristics and managements. J. Korean Neurosurg. Soc. 44, 116–123. doi: 10.3340/jkns.2008.44.3.116, PMID: 19096660 PMC2588299

[ref21] Passero S. G. Calchetti B. Bartalini S. (2005). Intracranial bleeding in patients with Vertebrobasilar Dolichoectasia. Stroke 36, 1421–1425. doi: 10.1161/01.STR.0000172311.64662.9c 15976311

[ref22] Passero S. G. Rossi S. (2008). Natural history of vertebrobasilar dolichoectasia. Neurology 70, 66–72. doi: 10.1212/01.wnl.0000286947.89193.f3 18166708

[ref23] Peng F. Fu M. Xia J. Niu H. Liu L. Feng X.. (2022). Quantification of aneurysm wall enhancement in intracranial fusiform aneurysms and related predictors based on high-resolution magnetic resonance imaging: a validation study. Ther. Adv. Neurol. Disord. 15:17562864221105342. doi: 10.1177/17562864221105342, PMID: 35847373 PMC9280813

[ref24] Quan K. Song J. Yang Z. Wang D. An Q. Huang L.. (2019). Validation of wall enhancement as a new imaging biomarker of Unruptured cerebral aneurysm. Stroke 50, 1570–1573. doi: 10.1161/STROKEAHA.118.024195, PMID: 31035900

[ref25] Raghuram A. Varon A. Roa J. A. Ishii D. Lu Y. Raghavan M. L.. (2021). Semiautomated 3D mapping of aneurysmal wall enhancement with 7T-MRI. Sci. Rep. 11:18344. doi: 10.1038/s41598-021-97727-0, PMID: 34526579 PMC8443635

[ref26] Raghuram A. Varon A. Sanchez S. Ishii D. Wu C. Magnotta V. A.. (2022). Topographical analysis of Aneurysm Wall enhancement with 3-dimensional mapping. Stroke Vasc. Interv. Neurol. 2:e000309. doi: 10.1161/SVIN.121.000309 36061513 PMC9432773

[ref27] Roa J. A. Zanaty M. Osorno-Cruz C. Ishii D. Bathla G. Ortega-Gutierrez S.. (2020). Objective quantification of contrast enhancement of unruptured intracranial aneurysms: a high-resolution vessel wall imaging validation study. J. Neurosurg. 134, 862–869. doi: 10.3171/2019.12.JNS192746, PMID: 32032948 PMC7415549

[ref28] Sabotin R. P. Varon A. Roa J. A. Raghuram A. Ishii D. Nino M.. (2021). Insights into the pathogenesis of cerebral fusiform aneurysms: high-resolution MRI and computational analysis. J. NeuroInterventional Surg. 13, 1180–1186. doi: 10.1136/neurintsurg-2020-017243, PMID: 33632878

[ref29] Sacho R. H. Saliou G. Kostynskyy A. Menezes R. Tymianski M. Krings T.. (2014). Natural history and outcome after treatment of Unruptured Intradural fusiform aneurysms. Stroke 45, 3251–3256. doi: 10.1161/STROKEAHA.114.006292, PMID: 25205312

[ref30] Samaniego E. A. Roa J. A. Hasan D. (2019). Vessel wall imaging in intracranial aneurysms. J. Neuro Interventional Surg. 11, 1105–1112. doi: 10.1136/neurintsurg-2019-014938 31337731

[ref31] Shimonaga K. Matsushige T. Ishii D. Sakamoto S. Hosogai M. Kawasumi T.. (2018). Clinicopathological insights from Vessel Wall imaging of Unruptured intracranial aneurysms. Stroke 49, 2516–2519. doi: 10.1161/STROKEAHA.118.021819, PMID: 30355091

[ref32] Turjman A. S. Turjman F. Edelman E. R. (2014). Role of fluid dynamics and inflammation in intracranial aneurysm formation. Circulation 129, 373–382. doi: 10.1161/CIRCULATIONAHA.113.001444, PMID: 24446407 PMC4371596

[ref33] Wang X. Zhu C. Leng Y. Degnan A. J. Lu J. (2019). Intracranial Aneurysm Wall enhancement associated with aneurysm rupture: a systematic review and Meta-analysis. Acad. Radiol. 26, 664–673. doi: 10.1016/j.acra.2018.05.005, PMID: 29908979

