# Supplementary material for: Three-dimensional aneurysm wall enhancement in fusiform intracranial aneurysms is associated with aneurysmal symptoms
Source: Front Neurosci. 2023 May 5;17:1171946. doi: 10.3389/fnins.2023.1171946 (PMC10196058; doi:10.3389/fnins.2023.1171946)
Supplement: Supplementary file 1 [file Table_1.DOCX]

**Supplemental Table 1**. Imaging parameters of 3D T1W sequence.

| 3D T1W | TR/TE  (ms) | Flip angle ° | field of view (mm^2^) | matrix | slice thickness (mm) | inplane resolution  (mm^2^) | acquisition time |
| --- | --- | --- | --- | --- | --- | --- | --- |
| VISTA | 800/20 | 180 | 160×160 | 256×256 | 0.6 | 0.70×0.70 | 5´34" |
| CUBE | 600/20 | 180 | 160×180 | 256×256 | 0.6-0.7 | 0.70×0.70 | 4´04" |
| SPACE | 800/22 | 180 | 180×180 | 256×256 | 0.7 | 0.70×0.70 | 5´30" |

VISTA, volumetric isotropic turbo spin echo acquisition sequence; SPACE, sampling perfection with application-optimized contrast using different flip angle evolution; TE, echo time; TR, repetition time.
